# Microwave-assisted nucleophilic fluorination: a facile approach to the synthesis of 6′,6′-*gem*-difluorinated carbocyclic nucleosides

**DOI:** 10.3389/fchem.2026.1789531

**Published:** 2026-03-23

**Authors:** Gang Huang, Xiankai Li, Deyun Cui, Wubin Zhi, Peng Qi, Xiaofei Hao, Peng Liu

**Affiliations:** 1 School of Chemical Engineering, Zhengzhou University, Zhengzhou, China; 2 Institute of Chemistry, Henan Academy of Sciences, Zhengzhou, China; 3 Quality Inspection and Analysis Testing Research Center, Henan Academy of Sciences, Zhengzhou, China

**Keywords:** antiviral agent, aristeromycin, carbocyclic nucleosides, microwave-assisted synthesis, nucleophilic fluorination

## Abstract

Nucleophilic fluorination of a sterically hindered cyclopentanone intermediate under microwave irradiation is established to construct the key 6′,6′-*gem*-difluorocarbocyclic scaffold, which provides a practical approach to the synthesis of 6′,6′-*gem*-difluorinated carbocyclic nucleosides. The requisite cyclopentanone was accessed through the optimized oxidative cleavage of a methylenecyclopentane precursor derived from an intramolecular radical cyclization. This concise strategy shortens the synthetic steps and enables the expansion of nucleobase diversity for 6′,6′-*gem*-difluorinated carbocyclic nucleosides.

## Introduction

**FIGURE 1:**
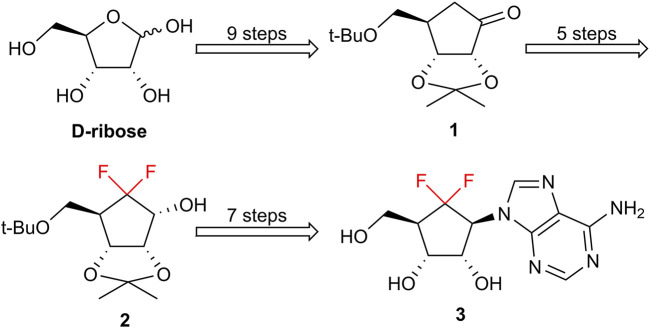
Reported synthetic approach to 6′,6′-*gem*-difluoroaristeromycin.

Recently, Kim et al. (2017), Yoon et al. (2019) reported 6′,6′-*gem*-difluoroaristeromycin (DFA, **3**) exhibiting the highest potent antiviral activity against several RNA viruses, including MERS-CoV, SARS-CoV, chikungunya virus, and Zika virus (Kim et al., 2017; Yoon et al., 2019). Preliminary studies suggested that it functions as dual-target antiviral agent by inhibiting both viral RdRp and host S-adenosylhomocysteine (SAH) hydrolase, highlighting its potential as a lead structure for the development of broad-spectrum antivirals (Ogando et al., 2021). Carbocyclic nucleoside analogs (CNAs), in which the furanose oxygen is replaced by a methylene group, represent a distinctive and valuable class in nucleoside chemistry (Bessières et al., 2015; Boutureira et al., 2013; Ojeda-Porras et al., 2022). However, the discovery and development of carbocyclic nucleoside antivirals are frequently limited by synthetic complexity. The reported synthesis of DFA from *D*-ribose requires up to 21 steps, while the most challenging step was the introduction of the CF_2_ group via electrophilic fluorination (Figure 1) (Yoon et al., 2019). In this article, we report an alternative synthetic route to construct the key 6′,6′-*gem*-difluorocarbocyclic scaffold by microwave-assisted nucleophilic fluorination of a sterically hindered cyclopentanone.

## Materials and methods

Detailed experimental procedures and compound characterization data are provided in the Supplementary Material.

## Results and discussion

**FIGURE 2:**
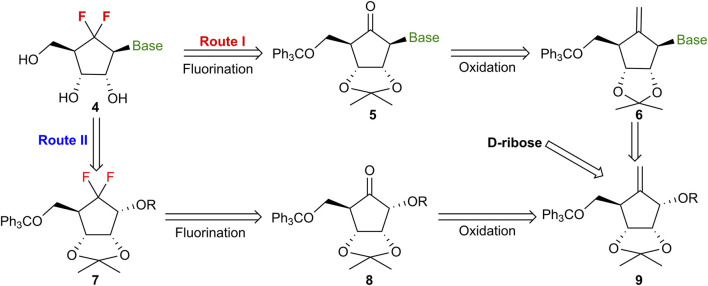
Retrosynthetic analysis of 6′,6′-*gem*-difluorocarbocyclic nucleosides.

As outlined in Figure 2, installation of the 6′,6′-*gem*-difluorocarbocyclic motifs can be accomplished in a single step through nucleophilic fluorination of a cyclopentanone, according to prior reports of diethylaminosulfur trifluoride (DAST)-mediated difluorination of ketones (Gumina et al., 2001; Shen and Hong, 2014; Wang et al., 2007). Two synthetic strategies were explored. In Route I, nucleobase construction precedes oxidative cleavage and subsequent difluorination; however, significant steric congestion from the nucleobase and protecting groups was expected to impede these downstream transformations. Conversely, Route II employs a strategy where the difluorination of the polyhydroxylated cyclopentanone (**8**) is executed prior to the installation of the nucleobase, a sequence designed to mitigate steric hindrance during the fluorination step. A significant obstacle inherent in this approach involves the *de novo* construction of cyclopentanones possessing multiple chiral hydroxyl substituents. To address this, our strategy leverages the oxidative cleavage of a methylenecyclopentane precursor (**9**), which is readily accessed from *D*-ribose through an intramolecular radical cyclization of an alkyne intermediate (Gaudino and Craig, 1990; Takagi et al., 2005).

**FIGURE 3:**
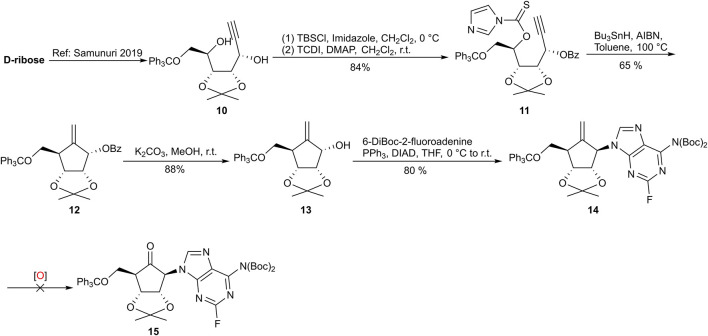
Preliminary synthesis of **15** via the introduction of the base moiety, followed by oxidation.

As illustrated in Route I (Figure 3), diol **10** was synthesized from *D*-ribose with high stereoselectivity (Samunuri et al., 2019), notably obviating the need for column chromatography through recrystallization from a mixture of methyl tert-butyl ether and n-hexane. Subsequent selective protection of the α-hydroxyl group with a benzoyl (Bz) group, followed by treatment with thiocarbonyldiimidazole (TCDI), yielded the corresponding imidazole xanthate as a radical precursor **11**. The key intramolecular radical cyclization with terminal alkyne was then executed under Barton–McCombie deoxygenation conditions (Crich and Quintero, 1989). This transformation is postulated to proceed via a chair-like transition state, where the chemical yield and diastereoselectivity are primarily dictated by steric interactions between the protecting groups (Gaudino and Craig, 1990). Under optimized conditions, the desired diastereoisomer **12** was isolated in 65% yield. Removal of the benzoyl group in carbocycle **12** with potassium carbonate in methanol, followed by the introduction of 6-di-tert-butoxycarbonyl-2-fluoroadenine via the Mitsunobu reaction, afforded the desired nucleoside **14** in 80% yield over two steps.

Efforts were subsequently directed toward the oxidative cleavage of the exocyclic alkene to produce cyclopentanone **15**. However, this transformation proved formidable as exhaustive optimization of oxidative conditions yielded no success. Conventional protocols, including ozonolysis and NaIO_4_-mediated oxidation (Hanessian et al., 2005; Sakaguchi et al., 2007), resulted exclusively in either the recovery of starting material or non-specific decomposition. Interestingly, when subjected to Lemieux–Johnson conditions (K_2_OsO_4_/NaIO_4_) at room temperature for 36 h, the reaction stalled at the dihydroxylation stage, providing only the corresponding diol intermediate, detected by HRMS. This observation suggests that the subsequent oxidative cleavage of the resulting diol was significantly impeded, likely due to the inherent steric environment.

**FIGURE 4:**
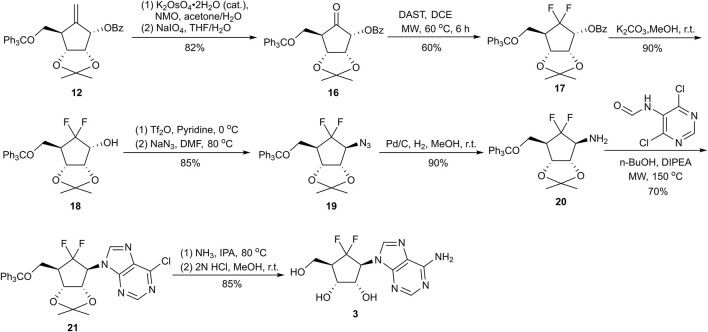
Facile synthesis of 6′,6′-*gem*-difluoroaristeromycin **3**.

To circumvent the aforementioned synthetic hurdles, we pivoted our strategy to investigate oxidative cleavage and fluorination prior to the installation of the nucleobase (Route II, Figure 4). Initial attempts utilizing ozonolysis or RuCl_3_/NaIO_4_-mediated oxidation again proved fruitless, yielding complex mixtures. While a one-pot treatment with Lemieux–Johnson conditions at room temperature for 36 h did afford cyclopentanone **16**, it provided only a modest 48% yield. The sluggish conversion of alkene **12** suggested that the resulting ketone **16** was prone to oxidative degradation during the protracted reaction time. To mitigate this instability, a stepwise protocol was developed: initial dihydroxylation under optimized Upjohn conditions (K_2_OsO_4_/NMO) proceeded quantitatively, followed by a discrete NaIO_4_-mediated oxidative cleavage in THF/H_2_O. This refined two-step sequence produced ketone **16** in an excellent 82% combined isolated yield.

Attention was then directed toward the installation of the *gem*-difluoride moiety at the 6′-position. *N,N*-dialkylaminosulfur trifluoride reagents, such as DAST and BAST, are well-recognized deoxofluorinating agents for converting ketones into CF_2_ groups (Kaźmierczak and Bilska-Markowska, 2021; Singh and Shreeve, 2002). However, initial attempts to fluorinate ketone **16** via conventional thermal heating with either reagent proved ineffective (Table 1, entries 1–2). Extended reaction durations resulted in sluggish conversion, characterized by substantial recovery of the starting material and the concomitant formation of polar byproducts. We attributed this lack of reactivity to the significant steric hindrance surrounding the carbonyl center, which imposed a high activation energy barrier that could not be effectively overcome by conventional heating without triggering competitive thermal decomposition. To circumvent this recalcitrance, we utilized microwave (MW) irradiation. The superiority of MW in this transformation stems from its capacity for rapid, uniform, and volumetric heating, which provides the precise energy required to facilitate the deoxofluorination while significantly reducing reaction times (Furuya et al., 2005; Kalar et al., 2023). Finally, MW irradiation with DAST at 60 °C for 6 h proved to be the optimal condition, yielding the desired *gem*-difluoro compound **17** in 50% isolated yield, along with 36% recovered starting material (entry 4). Notably, escalating the temperature to 80 °C or increasing the DAST loading failed to enhance conversion, instead resulting in extensive decomposition and the formation of an intractable dark mixture (entries 5–6). Notwithstanding the volume constraints of the MW reactor, the protocol was successfully implemented on a gram-scale, delivering compound **17** in an improved 60% isolated yield after column chromatography purification (entry 7).

**TABLE 1:** Nucleophilic fluorination of 16^a^.

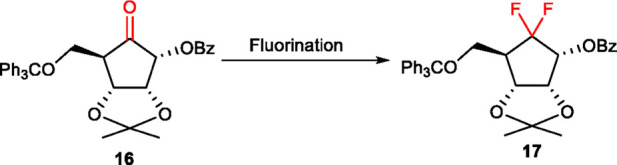			
Entry	Reagent (equiv.)	Temperature and time	Results^b^
1	DAST (20)	25 °C–40 °C, 48 h	**16** (80%), **17** (10%)
2	BAST (10)	80 °C, 16 h	**16** (86%), **17** (6%)
3	DAST (neat)	25 °C–60 °C, 48 h	**16** (35%), **17** (34%)
4	DAST (10)	MW, 60 °C, 6 h	**16** (40%), **17** (46%)
5	DAST (10)	MW, 80 °C, 2 h	**16** (20%), **17** (14%)
6	DAST (20)	MW, 60 °C, 6 h	**16** (33%), **17** (45%)
7^c^	DAST (10)	MW, 60 °C, 6 h	**16** (20%), **17** (65%)

^a^ Reactions were carried out with **16** (50 mg) in DCE (0.2 M).

^b^ Monitored by HPLC (254 nm).

^c^ Reaction scale: **16** (1.0 g) in DCE (10 mL).

The synthesis proceeded with the removal of the benzoyl protecting group in **17** using potassium carbonate in methanol to afford cyclopentanol **18**. Initial attempts to install the nucleobase directly onto the carbocyclic scaffold via Mitsunobu reaction or S_N_2 substitution under alkaline conditions were unproductive (Kim et al., 2017; Yoon et al., 2019). Consequently, we adopted an alternative strategy, inspired by the work of Jeong and co-workers, to access cyclopentylamine **20** (Figure 4). Azide **19** was efficiently prepared through a two-step sequence involving the conversion of alcohol **18** to its corresponding triflate, followed by nucleophilic displacement with sodium azide in DMF. Subsequent catalytic hydrogenation of **19** yielded amine **20**, which served as a versatile platform for nucleobase construction. Utilizing this key intermediate, we successfully achieved the synthesis of DFA, a potent broad-spectrum anti-RNA virus agent. Amine **20** was condensed with *N*-(4,6-dichloropyrimidin-5-yl)formamide in n-butanol at 150 °C under microwave irradiation, facilitating the rapid formation of nucleoside **21**. Subsequent amination with saturated ammonia in isopropanol (IPA), followed by global deprotection with 2 N HCl, afforded the target adenosine analog **3** in an impressive 85% isolated yield over two steps. Notably, this optimized route expedites the total synthesis of 6′,6′-*gem* difluorinated aristeromycin analogs by leveraging a direct nucleophilic fluorination strategy, offering a more concise alternative to previously established methodologies.

**FIGURE 5:**
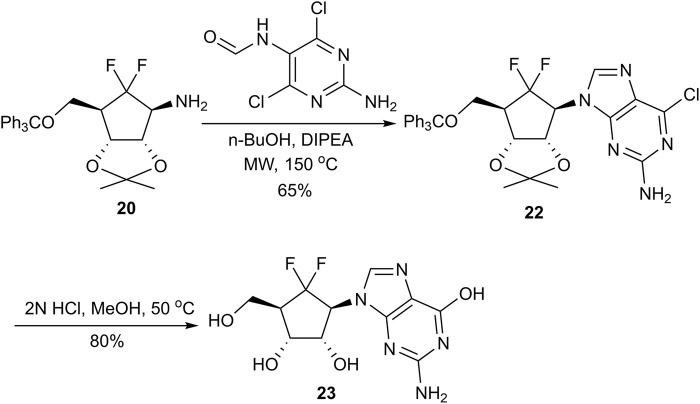
Synthesis of 6′,6′-difluorocarbocyclic nucleoside analog **23**.

Expanding the scope of this methodology, we also synthesized the 6′,6′-difluorocarbocyclic guanine analog **23** (Figure 5). Intermediate **20** was subjected to cyclization with *N*-(2,4-diamino-6-chloropyrimidin-5-yl)formamide under microwave irradiation to yield the chloroguanine intermediate **22**. Subsequent acidic hydrolysis of **22** successfully yielded the target. This successful diversification further demonstrates the versatility for accessing various 6′,6′-*gem*-difluorinated carbocyclic nucleosides.

**FIGURE 6:**
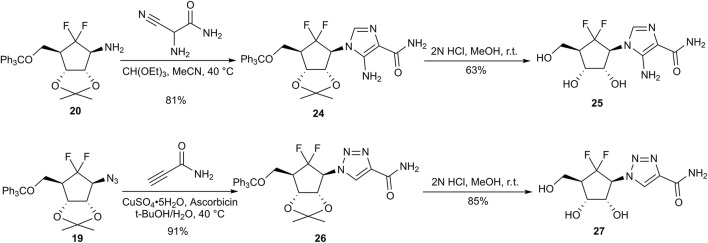
Synthesis of 6′,6′-difluorocarbocyclic nucleoside analogs **25** and **27**.

Given that imidazole- and triazole-based nucleoside analogs possess a broad spectrum of biological activities (An and Rhee, 2003; Cho et al., 2006; Nascimento et al., 2024), we further extended our synthetic platform to include these scaffolds. Imidazole carboxamide **25** was successfully synthesized via the cyclization of amine **20** with 2-amino-2-cyanoacetamide, followed by acidic deprotection. In a parallel approach, azide **19** was subjected to a copper-catalyzed azide–alkyne cycloaddition (CuAAC) with propiolamide, producing triazole carboxamide **26** in quantitative yield. Subsequent acid-mediated deprotection yielded the final compound **27** (Figure 6). Although preliminary antiviral evaluation of these specific analogs revealed no detectable activity, the synthetic strategy described herein establishes an efficient and robust protocol for constructing the 6′,6′-*gem*-difluorocarbocyclic framework. This methodology enables rapid nucleobase diversification and provides a versatile foundation for future structure–activity relationship (SAR) studies and the discovery of novel antiviral lead structures.

## Conclusion

In summary, we have developed an efficient and scalable synthetic route to the key, 6′,6′-*gem* difluorocarbocyclic scaffold, providing a versatile platform for the structural diversification of 6′,6′-*gem*-difluorinated carbocyclic nucleoside analogs. A robust protocol for the construction of highly functionalized, multi-hydroxylated cyclopentanones was established via the oxidative cleavage of methylenecyclopentanes, which are readily accessed through an intramolecular radical cyclization of alkyne precursors. Leveraging this strategy, we successfully synthesized a series of 6′,6′-*gem* difluorinated aristeromycin derivatives, including imidazole- and triazole-based analogs. Although these specific derivatives did not exhibit detectable antiviral activity in preliminary assays, the established methodology significantly expedites access to this complex carbocyclic framework. Ongoing efforts in our laboratory are focused on the further structural optimization of the adenosine core to enhance antiviral potency and explore broader biological applications.

## Data Availability

The original contributions presented in the study are included in the article/Supplementary Material; further inquiries can be directed to the corresponding author.
