# Supplementary material for: Microwave-assisted nucleophilic fluorination: a facile approach to the synthesis of 6′,6′-gem-difluorinated carbocyclic nucleosides
Source: Front Chem. 2026 Mar 23;14:1789531. doi: 10.3389/fchem.2026.1789531 (PMC13051287; doi:10.3389/fchem.2026.1789531)
Supplement: Supplementary file 1 [file Supplementaryfile1.docx]

**An Alternative Approach to the Synthesis of 6′,6′-gem-Difluorinated Aristeromycin Analogues via Nucleophilic Fluorination**

**EXPERIMENTAL SECTION**

**Chemical Synthesis.
General Methods.**
^1^H NMR (400 MHz) and ^13^C NMR (100 MHz) spectra were recorded on a Quantum 400 spectrometer. Chemical shifts (*δ*) are reported in parts per million (ppm) relative to residual solvent signals, and coupling constants (*J*) are reported in hertz (Hz). High-resolution mass spectra (HRMS) were obtained using a Thermo Scientific Orbitrap Exploris 120 instrument. Crude products were purified by silica gel column chromatography (200-300 mesh). The purity of all final compounds was ≥95%, as determined by high-performance liquid chromatography (HPLC).

**Figure 1 Synthesis of 11**

***(R)-1-((4R,5S)-5-((S)-1-(benzoyloxy)prop-2-yn-1-yl)-2,2-dimethyl-1,3-dioxolan-4-yl)-2-(trityloxy)ethyl 1H-imidazole-1-carboxylate (11)***

Diol **10** (34.0 g, 74.1 mmol) (prepared according to Samunuri (2019)) was dissolved in anhydrous dichloromethane (340 mL) and cooled to 0 °C. Benzoyl chloride (10.9 g, 77.8 mmol) was added, followed by slow addition of triethylamine (11.3 g, 111 mmol). The reaction mixture was allowed to warm to room temperature and stirred for 6 h. The reaction was quenched by water (100 mL) and the organic layer was collected. The aqueous layer was extracted with dichloromethane (50 mL × 2). The combined organic layers were successively washed with 0.5 N HCl, saturated NaHCO_3_, brine, and then dried over anhydrous Na_2_SO_4_, filtered, and evaporated. The crude product was used directly in the next step. The residue was dissolved in dry DCM (86 mL), followed by addition of 4-dimethylaminopyridine (4.53 g, 37.1 mmol) and thiocarbonyldiimidazole (19.8 g, 111 mmol). The mixture was stirred at rt for 16 h. Removal of the solvent under reduced pressure, the residue was purified by silica gel column chromatography (EtOAc/n-hexane, 1:3) to afford **11** (41.9 g, 84% yield over two steps) as a white solid. ^1^H NMR (400 MHz, CDCl_3_): δ 8.06 (s, 1H), 7.86 (d, *J* = 7.7 Hz, 2H), 7.52 (t, *J* = 7.5 Hz, 1H), 7.41-7.34 (m, 8H), 7.31 (t, *J* = 7.7 Hz, 2H), 7.19 (dd, *J* = 5.2, 2.0 Hz, 8H), 6.97 (d, *J* = 1.6 Hz, 1H), 5.75 (dt, *J* = 8.5, 2.6 Hz, 1H), 5.64 (dd, *J* = 6.4, 2.2 Hz, 1H), 5.08 (dd, *J* = 8.4, 5.7 Hz, 1H), 4.62 (t, *J* = 6.0 Hz, 1H), 3.74 (ddd, *J* = 47.3, 11.4, 2.7 Hz, 2H), 2.40 (d, *J* = 2.1 Hz, 1H), 1.53 (s, 3H), 1.49 (s, 3H); ^13^C NMR (100 MHz, CDCl_3_): δ 181.8, 164.9, 143.6, 137.0, 133.6, 130.8, 129.7, 129.2, 128.6, 128.5, 127.9, 127.2, 117.7, 109.9, 86.9, 79.3, 75.7, 74.4, 63.3, 60.7, 27.3, 25.4; HRMS (ESI+): m/z calcd for C_40_H_37_N_2_O_6_S [M+H]^+^ 673.2372; found 673.2374.

***(3aR,4S,6R,6aR)-2,2-dimethyl-5-methylene-6-((trityloxy)methyl)tetrahydro-4H-cyclopenta[d][1,3]dioxol-4-yl benzoate (12)***

A mixture of **11** (5.0 g, 7.43 mmol), Bu_3_SnH (3.12 g, 14.8 mmol), AIBN (609 mg, 3.72 mmol) in dry toluene (150 mL) was heated at 100 °C for 4 h. The resulting mixture was cooled to rt and washed with 1 M aqueous KF (50 mL × 2), brine. The solvent was removed and the residue was purified by silica gel column chromatography (EtOAc/n-hexane, 1:10) to give **12** (2.64 g, 65% yield) and **12b** (0.70 g, 17% yield) as a white foam.

Compound **12**: ^1^H NMR (400 MHz, CDCl_3_): δ 8.17-8.13 (m, 2H), 7.58 (t, *J* = 7.4 Hz, 1H), 7.49-7.41 (m, 9H), 7.32 (t, *J* = 7.5 Hz, 6H), 7.23 (d, *J* = 7.1 Hz, 2H), 5.75 (dq, *J* = 6.3, 2.2 Hz, 1H), 5.38 (t, *J* = 1.7 Hz, 1H), 5.27 (d, *J* = 2.6 Hz, 1H), 4.96 (t, *J* = 5.8 Hz, 1H), 4.49 (d, *J* = 5.4 Hz, 1H), 3.27-3.14 (m, 2H), 2.91-2.84 (m, 1H), 1.36 (s, 3H), 1.28 (s, 3H); ^13^C NMR (100 MHz, CDCl_3_): δ 166.1, 148.2, 143.8, 133.2, 130.0, 128.8, 128.5, 128.0, 127.3, 111.7, 111.3, 87.3, 82.2, 78.6, 75.2, 65.6, 49.3, 26.8, 25.5; HRMS (ESI+): m/z calcd for C_36_H_34_O_5_Na [M+Na]^+^ 569.2304; found 569.2312.

Compound **12b**: ^1^H NMR (400 MHz, CDCl_3_): δ 8.10-8.03 (m, 2H), 7.59-7.51 (m, 1H), 7.48 (d, *J* = 7.4 Hz, 6H), 7.40 (t, *J* = 7.8 Hz, 2H), 7.28 (t, *J* = 7.7 Hz, 5H), 7.24 – 7.16 (m, 4H), 5.30 (dd, *J* = 5.3, 2.5 Hz, 1H), 5.12 (t, *J* = 2.7 Hz, 1H), 4.87-4.80 (m, 3H), 3.59 (t, *J* = 8.3 Hz, 1H), 3.35 (dd, *J* = 8.9, 5.8 Hz, 1H), 2.60 (d, *J* = 2.9 Hz, 1H), 1.28 (s, 3H), 1.21 (s, 3H).

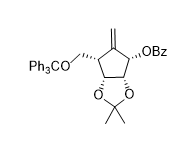


***(3aS,4S,6R,6aR)-2,2-dimethyl-5-methylene-6-((trityloxy)methyl)tetrahydro-4H-cyclopenta[d][1,3]dioxol-4-ol (13)***

To a solution of **12** (2.30 g, 4.21 mmol) in anhydrous MeOH (25 mL) was added K_2_CO_3_ (581 mg, 4.21 mmol) and the mixture was stirred at room temperature overnight. The reaction was evaporated and the residue was purified by silica gel column chromatography (EtOAc/n-hexane, 1:4) to give **13** (1.73 g, 88% yield) as a foam. ^1^H NMR (400 MHz, CDCl_3_): δ 7.41-7.36 (m, 6H), 7.32-7.27 (m, 5H), 7.25-7.20 (m, 4H), 5.39-5.34 (m, 1H), 5.19-5.14 (m, 1H), 4.72-4.65 (m, 1H), 4.62 (t, *J* = 5.8 Hz, 1H), 4.44 (d, *J* = 5.6 Hz, 1H), 3.19-3.10 (m, 2H), 2.69-2.66 (m, 1H), 2.34 (d, *J* = 10.5 Hz, 1H, OH), 1.40 (s, 3H), 1.32 (s, 3H); ^13^C NMR (100 MHz, CDCl_3_): δ 152.8, 143.8, 128.8, 128.0, 127.2, 110.7, 110.6, 87.2, 81.2, 79.2, 74.0, 65.9, 49.7, 26.6, 24.9; HRMS (ESI+): m/z calcd for C_29_H_30_O_4_Na [M+Na]^+^ 465.2042; found 465.2073.

***tert-butyl (tert-butoxycarbonyl)(9-((3aS,4R,6R,6aR)-2,2-dimethyl-5-methylene-6-((trityloxy)methyl)tetrahydro-4H-cyclopenta[d][1,3]dioxol-4-yl)-2-fluoro-9H-purin-6-yl)carbamate (14)***

To a solution of compound **13** (340 mg, 0.770 mmol), and tert-butyl (tert-butoxycarbonyl)(2-fluoro-9H-purin-6-yl)carbamate (354 mg, 1.01 mmol), Ph_3_P (303 mg, 1.10 mmol) in 5.0 mL of anhydrous THF was added DIAD (230 mg, 1.10 mmol) at 0 °C under N_2_ atmosphere, and the mixture was stirred for 4 h at room temperature. The reaction mixture was adsorbed on silica gel and then purified by silica gel column chromatography (EtOAc/n-hexane, 1:6) to give compound **14** (479 mg, 80% yield). ^1^H NMR (400 MHz, CDCl_3_): δ 7.96 (s, 1H), 7.46 (d, *J* = 7.8 Hz, 7H), 7.32-7.24 (m, 8H), 5.40 (d, *J* = 5.7 Hz, 1H), 4.90 (t, *J* = 5.9 Hz, 1H), 4.67 (dd, *J* = 6.4, 3.1 Hz, 1H), 4.55 (s, 1H), 3.57-3.43 (m, 2H), 3.15-3.07 (m, 1H), 1.62 (s, 3H), 1.48 (s, 18H), 1.36 (s, 3H); ^19^F NMR (376 MHz, CDCl_3_): δ -49.99; HRMS (ESI+): m/z calcd for C_44_H_48_FN_5_O_7_Na [M+Na]^+^ 800.3435; found 800.3430.

^19^F NMR (376 MHz, CDCl_3_)

***(3aR,4R,6R,6aR)-2,2-dimethyl-5-oxo-6-((trityloxy)methyl)tetrahydro-4H-cyclopenta[d][1,3]dioxol-4-yl benzoate (16)***

Methanesulfonamide (1.56 g, 16.5 mmol) was dissolved in acetone/water (60 mL, 3:1), followed by addition of N-methylmorpholine N-oxide (1.83 g, 16.5 mmol) and K_2_OsO_4_·2H_2_O (68.3 mg, 0.22 mmol). The mixture was stirred at room temperature for 30 min. A suspension of **12** (6.0 g, 11.0 mmol) in acetone/water (36 mL, 3:1) was added, and the reaction was stirred at room temperature for 48 h. Water (100 mL) was added and the mixture was extracted with EtOAc (100 mL × 3). The combined organic layers were washed with brine, concentrated. The residue was dissolved in THF/water (60 mL, 1:1), followed by addition of NaIO_4_ (7.1 g, 33.0 mmol). The mixture was stirred vigorously at room temperature for 6 h. The aqueous layer was extracted with EtOAc (50 mL × 3), and the combined organic layers were washed with brine, dried over anhydrous Na_2_SO_4_, filtered, and concentrated. Purification by silica gel column chromatography (EtOAc/n-hexane, 1:3) afforded cyclopentanone **16** (4.80 g, 82% yield). ^1^H NMR (400 MHz, CDCl_3_): δ 8.19-8.14 (m, 2H), 7.58 (t, *J* = 7.5 Hz, 1H), 7.46 (t, *J* = 7.7 Hz, 2H), 7.34 (dt, *J* = 15.0, 7.4 Hz, 12H), 7.24 (dd, *J* = 7.2, 1.6 Hz, 3H), 5.81 (d, *J* = 5.7 Hz, 1H), 5.09 (t, *J* = 5.6 Hz, 1H), 4.68 (dd, *J* = 5.4, 2.1 Hz, 1H), 3.66 (dd, *J* = 9.0, 3.5 Hz, 1H), 3.29 (dd, *J* = 9.1, 3.2 Hz, 1H), 2.47 (q, *J* = 3.0 Hz, 1H), 1.39 (s, 3H), 1.34 (s, 3H); ^13^C NMR (100 MHz, CDCl_3_): δ 209.8, 165.7, 143.2, 133.6, 130.3, 129.2, 128.7, 128.6, 128.2, 127.5, 112.2, 87.8, 78.1, 75.4, 62.6, 53.5, 27.3, 25.7; HRMS (ESI+): m/z calcd for C_35_H_33_O_6_Na [M+Na]^+^ 571.2097; found 571.2100.

***(3aR,4R,6R,6aR)-5,5-difluoro-2,2-dimethyl-6-((trityloxy)methyl)tetrahydro-4H-cyclopenta[d][1,3]dioxol-4-yl benzoate (17)***

To a solution of **16** (1.0 g, 1.83 mmol) in dry 1,2-dichloroethane (10 mL) was added diethylaminosulfur trifluoride (2.4 mL, 18.3 mmol) at room temperature and the mixture was stirred under MW/ 60 °C for 6 h. The reaction mixture was cooled and slowly poured into ice-cold saturated NaHCO_3_ solution (30 mL). The aqueous layer was extracted with CH_2_Cl_2_ (10 mL × 3). The combined organic layers were washed with saturated NaHCO_3_ and brine, dried over anhydrous Na_2_SO_4_, filtered, and concentrated. Purification by silica gel column chromatography (EtOAc/n-hexane, 1:5) gave **17** (0.44 g, 60% yield of conversion) and recovery of **16** (270 mg). ^1^H NMR (400 MHz, CDCl_3_): δ 8.16 (d, *J* = 7.6 Hz, 2H), 7.58 (d, *J* = 7.5 Hz, 1H), 7.46 (d, *J* = 7.9 Hz, 8H), 7.34 (t, *J* = 7.7 Hz, 6H), 7.28-7.24 (s, 3H), 5.61 (dt, *J* = 14.2, 7.1 Hz, 1H), 4.97-4.88 (m, 1H), 4.42 (t, *J* = 4.6 Hz, 1H), 3.53 (q, *J* = 3.9 Hz, 1H), 3.33 (t, *J* = 6.8 Hz, 1H), 2.71-2.57 (m, 1H), 1.40 (s, 3H), 1.26 (s, 3H); ^19^F NMR (376 MHz, CDCl_3_): δ -104.97 (dt, *J* = 240.4, 14.4 Hz), -111.63 (d, *J* = 240.2 Hz); ^13^C NMR (100 MHz, CDCl_3_): δ 165.4, 143.4, 133.5, 130.3, 128.8, 128.6, 128.2, 128.1, 127.4, 126.8 (dd, *J* = 253.8, 253.8 Hz), 112.3, 88.0, 79.8, 75.1 (d, *J* = 7.0 Hz), 74.3 (dd, *J* = 28.5, 16.6 Hz), 59.5, 49.3 (t, *J* = 20.5 Hz), 26.1, 24.7; HRMS (ESI+): m/z calcd for C_35_H_32_F_2_O_5_Na [M+Na]^+^ 593.2116; found 593.2119.

^19^F NMR (376 MHz, CDCl_3_)

***(3aS,4R,6R,6aR)-5,5-difluoro-2,2-dimethyl-6-((trityloxy)methyl)tetrahydro-4H-cyclopenta[d][1,3]dioxol-4-ol (18)***

To a solution of **17** (900 mg, 1.58 mmol) in anhydrous MeOH (10 mL) was added K_2_CO_3_ (218 mg, 1.58 mmol) and the mixture was stirred at room temperature overnight. The reaction was evaporated and the residue was purified by silica gel column chromatography (EtOAc/n-hexane, 1:4) to give **18** (667 mg, 90% yield). ^1^H NMR (400 MHz, CDCl_3_): δ 7.38 (d, *J* = 7.5 Hz, 6H), 7.31 (t, *J* = 7.5 Hz, 6H), 7.24 (d, *J* = 1.8 Hz, 3H), 4.64-4.60 (m, 1H), 4.53-4.43 (m, 1H), 4.38-4.36 (m, 1H), 3.46 (dd, *J* = 9.8, 3.2 Hz, 1H), 3.24 (dd, *J* = 9.7, 3.9 Hz, 1H), 2.87 (d, *J* = 10.5 Hz, 1H), 2.49-2.41 (m, 1H), 1.47 (s, 3H), 1.31 (s, 3H); ^19^F NMR (376 MHz, CDCl_3_): δ -107.27 (dt, *J* = 240.6, 15.9 Hz), -114.07 (dd, *J* = 240.6, 7.5 Hz); ^13^C NMR (100 MHz, CDCl_3_): δ 143.2, 128. 6, 128.0, 127.3, 126.8 (dd, *J* = 254.3, 254.4 Hz), 111.4, 87.9, 79.1 (d, *J* = 5.3 Hz), 75.3 (d, *J* = 9.9 Hz), 73.5 (dd, *J* = 26.5, 19.1 Hz), 59.5 (dd, *J* = 7.9, 4.4 Hz), 48.7 (t, *J* = 20.9 Hz), 25. 9, 24.2; HRMS (ESI+): m/z calcd for C_28_H_28_F_2_O_4_Na [M+Na]^+^ 489.1853; found 489.1850.


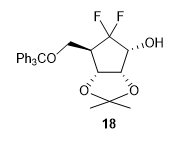

^19^F NMR (376 MHz, CDCl_3_)

***(3aS,4S,6R,6aR)-4-azido-5,5-difluoro-2,2-dimethyl-6-((trityloxy)methyl)tetrahydro-4H-cyclopenta[d][1,3]dioxole (19)***

To a solution of **18** (500 mg, 1.07 mmol) in pyridine (5.0 mL) at 0 °C was added triflic anhydride (0.36 mL, 2.14 mmol) dropwise, and the mixture was stirred for 0.5 h. The reaction was quenched with water (15 mL) and extracted with EtOAc (15 mL × 3). The combined organic layers were washed with 0.5 N HCl twice, saturated NaHCO_3_, and brine, dried over anhydrous Na_2_SO_4_, filtered, and concentrated. The residue was dried under vacuum and then dissolved in dry DMF (5.0 mL), followed by addition of NaN_3_ (209 mg, 3.21 mmol). The mixture was stirred at 80 °C for 4 h and quenched with water (15 mL). The mixture was extracted with EtOAc (10 mL × 3) and the combined organic layers were washed with saturated NaHCO_3_ and brine, dried over anhydrous Na_2_SO_4_, filtered, and concentrated. Purification by silica gel column chromatography (EtOAc/n-hexane, 1:5) gave azide **19** (447 mg, 85% yield over two steps). ^1^H NMR (400 MHz, CDCl_3_): δ 7.46 (d, *J* = 7.7 Hz, 6H), 7.31 (t, *J* = 7.6 Hz, 6H), 7.25 (d, *J* = 6.9 Hz, 3H), 4.27 (dt, *J* = 23.1, 6.7 Hz, 2H), 3.95 (dd, *J* = 15.2, 7.3 Hz, 1H), 3.47 (t, *J* = 8.6 Hz, 1H), 3.29 (t, *J* = 8.4 Hz, 1H), 2.73 (dt, *J* = 20.9, 6.5 Hz, 1H), 1.52 (s, 3H), 1.26 (s, 3H); ^19^F NMR (376 MHz, CDCl_3_): δ -100.04 (dt, *J* = 237.5, 7.7 Hz), -118.85 (dt, *J* = 237.6, 18.5 Hz); ^13^C NMR (100 MHz, CDCl_3_): δ 143.7, 128.8, 128.0, 127.3, 126.8 (dd, *J* = 254.2, 254.2 Hz), 113.6, 87.2, 79.6 (d, *J* = 6.7 Hz), 77.9 (d, *J* = 7.5 Hz), 69.0 (dd, *J* = 23.8, 18.8 Hz), 59.3 (d, *J* = 6.0 Hz), 50.3 (t, *J* = 19.8 Hz), 27.1, 24.8; HRMS (ESI+): m/z calcd for C_28_H_27_F_2_N_3_O_3_Na [M+Na]^+^ 514.1918; found 514.1926.


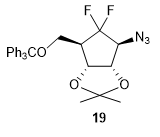

^19^F NMR (376 MHz, CDCl_3_)

***(3aS,4S,6R,6aR)-5,5-difluoro-2,2-dimethyl-6-((trityloxy)methyl)tetrahydro-4H-cyclopenta[d][1,3]dioxol-4-amine (20)***

To a suspension of **19** (300 mg, 0.610 mmol) in methanol (5.0 mL), 10% palladium on activated carbon (10% w/w) was added and stirred for 2 h at room temperature in a H₂ atmosphere (balloon). After filtration, the solvent was removed, and the residue (256 mg, 90% yield) was used for the next step without further purification. ^1^H NMR (400 MHz, CDCl_3_): δ 7.47 (d, *J* = 7.7 Hz, 7H), 7.31 (d, *J* = 7.4 Hz, 4H), 7.25 (d, *J* = 7.7 Hz, 4H), 4.20 (d, *J* = 6.0 Hz, 1H), 4.11 (t, *J* = 7.2 Hz, 1H), 3.42 (ddt, *J* = 25.3, 12.4, 6.8 Hz, 2H), 3.31 (dd, *J* = 9.3, 6.6 Hz, 1H), 2.74-2.65 (m, 1H), 1.52 (s, 3H), 1.27 (s, 3H); ^19^F NMR (376 MHz, CDCl_3_): δ -105.64 (dt, *J* = 233.4, 7.2 Hz), -123.41 (dt, *J* = 233.5, 21.1 Hz); ^13^C NMR (100 MHz, CDCl_3_): δ 143.8, 128.8, 127.9, 127.1, 127.3 (t, *J* = 261.4 Hz), 112.9, 87.0, 82.6 (d, *J* = 8.6 Hz), 77.6, 62.6 (t, *J* = 21.7 Hz), 59.7 (d, *J* = 6.0 Hz), 50.1 (t, *J* = 20.4 Hz), 27.2, 24.8; HRMS (ESI+): m/z calcd for C_28_H_29_F_2_NO_3_Na [M+Na]^+^ 488.2013; found 488.2013.


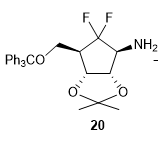

^19^F NMR (376 MHz, CDCl_3_)

***6-chloro-9-((3aS,4S,6R,6aR)-5,5-difluoro-2,2-dimethyl-6-((trityloxy)methyl)tetrahydro-4H-cyclopenta[d][1,3]dioxol-4-yl)-9H-purine (21)***

To a solution of **20** (50 mg, 0.107 mmol) in n-butanol (2.0 mL), *N*-(4,6-dichloropyrimidin-5-yl)formamide (103 mg, 0.535 mmol) and diisopropylethylamine (186 μL, 1.07 mmol) were added. The reaction mixture was placed under microwave irradiation at 150 °C for 5 h. The solvent was evaporated and the residue was purified with Prep-TLC (EtOAc/n-hexane, 1:3) to give **21** (46 mg, 70% yield). ^1^H NMR (400 MHz, CDCl_3_): δ 8.71 (s, 1H), 8.14 (s, 1H), 7.38 (d, *J* = 7.9 Hz, 6H), 7.20 (dd, *J* = 16.6, 7.3 Hz, 9H), 5.28 (dt, *J* = 23.5, 6.1 Hz, 1H), 5.04 (t, *J* = 7.4 Hz, 1H), 4.39 (t, *J* = 6.7 Hz, 1H), 3.43 (dt, *J* = 41.7, 8.9 Hz, 2H), 3.03-2.88 (m, 1H), 1.53 (s, 3H), 1.23 (s, 3H); ^19^F NMR (376 MHz, CDCl_3_): δ -103.89 (d, *J* = 235.1 Hz), -116.38 (dt, *J* = 235.5, 24.2 Hz); ^13^C NMR (100 MHz, CDCl_3_): δ 152.64, 152.54, 151.70, 143.84, 143.56, 131.54, 125.6 (t, *J* = 267.0 Hz), 114.7, 87.3, 78.5 (d, *J* = 7.5 Hz), 64.3 (t, *J* = 20.9 Hz), 59.2, 50.1 (t, *J* = 19.3 Hz), 27.4, 25.0; HRMS (ESI+): m/z calcd for C_33_H_30_ClF_2_N_4_O_3_ [M+H]^+^ 603.1974; found 603.1985.


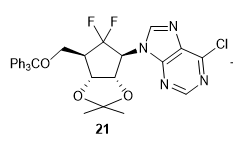

^19^F NMR (376 MHz, CDCl_3_)

***(1R,2S,3S,5R)-3-(6-amino-9H-purin-9-yl)-4,4-difluoro-5-(hydroxymethyl)cyclopentane-1,2-diol (3)***

To a solution of **21** (35 mg, 0.058 mmol) in saturated ammonia of IPA (5.0 mL) contained in a stainless bomb reactor and the reactor was heated to 80 °C for 15 h. The mixture was cooled to room temperature and evaporated. The residue was dissolved in MeOH (5.0 mL), followed by addition of 2 N HCl (2.0 mL) and the reaction mixture was stirred at rt for 6 h. Purification by preparative reversed-phase C18 column chromatography afforded the product **4** (15 mg, 85% yield) as white solid. ^1^H NMR (400 MHz, CD_3_OD): δ 8.27 (s, 1H), 8.21 (s, 1H), 5.34 (dt, *J* = 17.9, 10.0 Hz, 1H), 4.85-4.80 (m, 1H), 4.19-4.14 (m, 1H), 3.94-3.79 (m,2H), 2.73-2.61 (m, 1H); ^19^F NMR (376 MHz, CD_3_OD): δ -97.61 (d, *J* = 237.7 Hz), -115.53 (dt, *J* = 238.1, 18.5 Hz); ^13^C NMR (100 MHz, CD_3_OD): δ 157.3, 153.9, 151.8, 142.0, 125.1 (t, *J* = 258.8 Hz), 119.8, 72.9 (d, *J* = 7.5 Hz), 71.1, 64.0 (dd, *J* = 23.5 Hz), 58.8 (d, *J* = 10.8 Hz), 55.6 (t, *J* = 20.3 Hz); HRMS (ESI+): m/z calcd for C_11_H_14_F_2_N_5_O_3_ [M+H]^+^ 302.1065; found 302.1080.

^19^F NMR (376 MHz, CDCl_3_)

**6-chloro-9-((3aS,4S,6R,6aR)-5,5-difluoro-2,2-dimethyl-6-((trityloxy)methyl)tetrahydro-4H-cyclopenta[d][1,3]dioxol-4-yl)-9H-purin-2-amine (22)**

To a solution of **20** (50 mg, 0.107 mmol) in n-butanol (2.0 mL), *N*-(2-amino-4,6-dichloropyrimidin-5-yl)formamide (112 mg, 0.535 mmol) and diisopropylethylamine (186 μL, 1.07 mmol) were added. The reaction mixture was placed under microwave irradiation at 150 °C for 5 h. The solvent was evaporated and the residue was purified with Prep-TLC (EtOAc/n-hexane, 1:3) to give **22** (41 mg, 65% yield). ^1^H NMR (400 MHz, CDCl_3_): δ 7.80 (s, 1H), 7.40-7.38 (m, 6H), 7.26-7.16 (m, 9H), 5.10 (s, 2H), 5.06-5.05 (m, 1H), 4.91 (t, *J* = 7.3 Hz, 1H), 4.34 (t, *J* = 6.4 Hz, 1H), 3.41 (dt, *J* = 47.2, 8.8 Hz, 2H), 2.96- 2.87 (m, 1H), 1.53 (s, 3H), 1.24 (s, 4H); ^19^F NMR (376 MHz, CDCl_3_): δ -104.12 (d, *J* = 235.5 Hz), -116.50 (dt, *J* = 235.5, 24.3 Hz); ^13^C NMR (100 MHz, CDCl_3_): δ 159.4, 154.6, 151.8, 143.6, 140.7, 128.8, 128.0, 127.3, 125.6 (dd, *J* = 251.1, 254.6 Hz), 114.5, 87.2, 63.4 (t, *J* = 20.8 Hz), 59.3, 50.0 (t, *J* = 19.5 Hz), 27.4, 25.0; HRMS (ESI+): m/z calcd for C_33_H_31_ClF_2_N_5_O_3_ [M+H]^+^ 618.2083; found 618.2080.


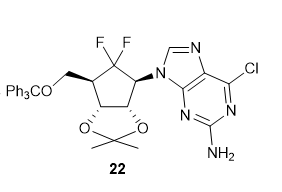

^19^F NMR (376 MHz, CDCl_3_)

***(1R,2S,3S,5R)-3-(2-amino-6-hydroxy-9H-purin-9-yl)-4,4-difluoro-5-(hydroxymethyl)cyclopentane-1,2-diol (22)***

To a stirred solution of **22** (35 mg, 0.057 mmol) in MeOH (2.0 mL), 2 N HCl (1.0 mL) was dropwise added and the reaction mixture was stirred at 50 °C for 2 h. Purification by preparative reversed-phase C18 column chromatography afforded the product **16** (14 mg, yield 80%) as white solid. ^1^H NMR (400 MHz, DMSO-*d*_6_) δ 10.66 (s, 1H), 7.81 (d, *J* = 2.5 Hz, 1H), 6.55 (s, 2H), 5.58 (d, *J* = 6.6 Hz, 1H), 5.29 (d, *J* = 3.5 Hz, 1H), 5.15-4.80 (m, 2H), 4.55-4.38 (m, 1H), 3.92 (s, 1H), 3.76-3.52 (m, 2H), 2.45-2.34 (m, 1H); ^19^F NMR (376 MHz, DMSO-d6) δ -73.47 (CF_3_COOH), -93.96 (d, *J* = 233.1 Hz), -113.37 (dt, *J* = 234.3, 16.0 Hz); ^13^C NMR (100 MHz, DMSO-*d*_6_) δ 156.8, 153.8, 152.3, 136.5, 125.4 (dd, *J* = 297.9, 257.1 Hz), 116.0, 71.1 (d, *J* = 7.8 Hz), 69.2, 60.9 (t, *J* = 21.6 Hz), 57.0 (d, *J* = 11.7 Hz), 54.3 (t, *J* = 18.7 Hz); HRMS (ESI+) m/z calcd for C_11_H_14_F_2_N_5_O_4_ [M+H]^+^ 318.1014, found 318.1007.


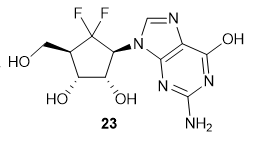

^19^F NMR (376 MHz, CDCl_3_)

***5-amino-1-((3aS,4S,6R,6aR)-5,5-difluoro-2,2-dimethyl-6-((trityloxy)methyl)tetrahydro-4H-cyclopenta[d][1,3]dioxol-4-yl)-1H-imidazole-4-carboxamide (24)***

To a solution of 2-amino-2-cyanoacetamide (32 mg, 0.322 mmol) in dry MeCN (2 mL) was added triethyl orthoformate (36 μL, 0.215 mmol) and the resulting mixture was stirred at 70 °C for 2 h. The mixture was cooled to room temperature, followed by addition of compound **20** (100 mg, 0.215 mmol). The reaction mixture was stirred at reflux for overnight and quenched by ice water (10 mL). The aqueous layer was extracted with EtOAc (10 mL × 3) and the combined organic layers were washed with brine, dried over anhydrous Na_2_SO_4_, filtered, and concentrated. Purification by silica gel column chromatography (EtOAc/n-hexane, 1:1) gave **24** (100 mg, 81% yield). ^1^H NMR (400 MHz, CDCl_3_) δ 7.47-7.38 (m, 6H), 7.33-7.21 (m, 6H), 7.21-7.10 (m, 4H), 6.61 (br, 1H), 5.50 (br, 1H), 5.20 (s, 2H), 4.86-4.59 (m, 1H), 4.57-4.44 (m, 1H), 4.27 (t, *J* = 6.4 Hz, 1H), 3.54-3.22 (m, 2H), 3.02-2.80 (m, 1H), 1.51 (s, 3H), 1.21 (s, 3H); ^19^F NMR (376 MHz, CDCl_3_) δ -103.41 (d, *J* = 235.5 Hz), -116.31 (dt, *J* = 235.5, 24.8 Hz); ^13^C NMR (100 MHz, CDCl_3_) δ 167.0, 143.5, 143.4, 128.7, 128.3 (d, *J* = 7.1 Hz), 128.0, 127.3, 125.9 (dd, *J* = 251.0 , 233.7 Hz), 114.6, 114.2, 87.2, 78.9 (d, *J* = 7.5 Hz), 77.0 (d, *J* = 8.9 Hz), 63.7 (t, *J* = 20.2 Hz), 59.1 (d, *J* = 6.3 Hz), 49.9 (t, *J* = 19.5 Hz), 27.1, 24.7; HRMS (ESI+) m/z calcd for C_32_H_33_F_2_N_4_O_4_ [M+H]⁺ 575.2470, found 575.2480.


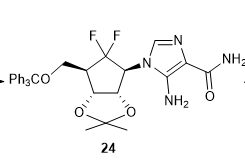

^19^F NMR (376 MHz, CDCl_3_)

***5-amino-1-((1S,3R,4R,5S)-2,2-difluoro-4,5-dihydroxy-3-(hydroxymethyl)cyclopentyl)-1H-imidazole-4-carboxamide (25)***

To a stirred solution of **24** (100 mg, 0.174 mmol) in MeOH (5.0 mL), 2 N HCl (2.0 mL) was dropwise added and the reaction mixture was stirred at 50 °C for 2 h. Purification by preparative reversed-phase C18 column chromatography afforded the product **25** (32 mg, yield 63%) as white solid. ^1^H NMR (400 MHz, CD_3_OD) δ 7.36 (s, 1H), 4.86-4.70 (m, 1H), 4.60-4.48 (m, 1H), 4.08 (s, 1H), 3.82 (dd, *J* = 15.4, 6.6 Hz, 2H), 2.60 (t, *J* = 15.5 Hz, 1H); ^19^F NMR (376 MHz, CD_3_OD) δ -97.68 (dt, *J* = 238.6, 12.5 Hz), -115.98 (dt, *J* = 238.4, 16.4 Hz); ^13^C NMR (100 MHz, CD_3_OD) δ 169.2, 146.5, 131.2, 125.3 (d, *J* = 257.5 Hz), 112.9, 72.6 (d, *J* = 7.8 Hz), 71.0, 63.2 (d, *J* = 21.8 Hz), 62.9, 58.7 (d, *J* = 10.7 Hz), 55.5 (t, *J* = 20.1 Hz); HRMS (ESI+) m/z calcd for C_10_H_15_F_2_N_4_O_4_ [M+H]^+^ 293.1061, found 293.1059.


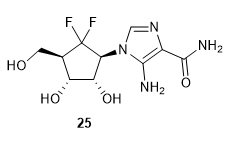

^19^F NMR (376 MHz, CDCl_3_)

***1-((3aS,4S,6R,6aR)-5,5-difluoro-2,2-dimethyl-6-((trityloxy)methyl)tetrahydro-4H-cyclopenta[d][1,3]dioxol-4-yl)-1H-1,2,3-triazole-4-carboxamide (26)***

To a solution of **19** (20 mg, 0.041 mmol) in tert-Butanol (1.0 mL)/water (0.5 mL) was added propiolamide (3.0 mg, 0.049 mmol), CuSO₄·5H₂O (2.0 mg, 0.008 mmol) and ascorbic acid (4.0 mg, 0.021 mmol). The mixture was stirred at 40 °C for overnight. The solvent was evaporated and the residue was purified with silica gel column chromatography (EtOAc/n-hexane, 1:2) to give **26** (21 mg, yield 91%). ^1^H NMR (400 MHz, CDCl_3_): δ 8.29 (s, 1H), 7.45 (d, *J* = 7.6 Hz, 7H), 7.34-7.23 (m, 8H), 7.11 (s, 1H), 5.93 (s, 1H), 5.22-5.09 (m, 2H), 4.40 (t, *J* = 6.3 Hz, 1H), 3.53-3.38 (m, 2H), 2.99-2.90 (m, 1H), 1.57 (s, 3H), 1.31 (s, 3H); ^19^F NMR (376 MHz, CDCl_3_): δ -102.74 (d, *J* = 235.0 Hz), -118.06 (dt, *J* = 235.0, 22.4 Hz); ^13^C NMR (100 MHz, CDCl_3_): δ 161.7, 143.4, 143.2, 128.7, 128.0, 127.3, 126.3, 124.90 (dd, J = 253.6, 253.6 Hz), 114.5, 87.2, 78.4 (d, *J* = 6.7 Hz), 77.2, 69.6 (t, *J* = 20.8 Hz), 58.9 (d, *J* = 6.1 Hz), 50.1 (t, *J* = 19.4 Hz), 27.2, 24.8; HRMS (ESI+): m/z calcd for C_31_H_30_F_2_N_4_O_4_Na [M+Na]^+^ 583.2133; found 583.2101.

^19^F NMR (376 MHz, CDCl_3_)

***1-((1S,3R,4R,5S)-2,2-difluoro-4,5-dihydroxy-3-(hydroxymethyl)cyclopentyl)-1H-1,2,3-triazole-4-carboxamide (27)***

To a stirred solution of **26** (20 mg, 0.035 mmol) in MeOH (2.0 mL), 2 N HCl (1.0 mL) was dropwise added and the reaction mixture was stirred at 50 °C for 2 h. Purification by preparative reversed-phase C18 column chromatography afforded the product **27** (8.5 mg, yield 85%). ^1^H NMR (400 MHz, CD_3_OD) δ 8.55 (s, 1H), 5.45- 5.36 (m, 1H), 4.74-4.70 (m, 1H), 4.13 (dd, *J* = 5.3, 2.9 Hz, 1H), 3.88-3.76 (m, 2H), 2.71-2.61 (m, 1H); ^19^F NMR (376 MHz, CD_3_OD) δ -97.07 (dt, *J* = 237.9, 12.8 Hz), -116.64 (dt, *J* = 238.0, 15.1 Hz); ^13^C NMR (100 MHz, CD_3_OD) δ 164.5, 144.0, 127.9, 124.6 (t, *J* = 259.0 Hz), 73.4 (d, *J* = 6.9 Hz), 71.1, 69.8 (dd, *J* = 24.6, 19.0 Hz), 58.6 (d, *J* = 11.0 Hz), 55.7 (t, *J* = 20.1 Hz); HRMS (ESI+) m/z calcd for C_9_H_12_F_2_N_4_O_4_Na [M+Na]^+^ 301.0724, found 301.0720.


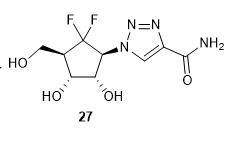

^19^F NMR (376 MHz, CDCl_3_)
